# G-Protein Coupled Receptor-Evoked Glutamate Exocytosis from Astrocytes: Role of Prostaglandins

**DOI:** 10.1155/2014/254574

**Published:** 2014-01-16

**Authors:** Corrado Cali, Jan Lopatar, Francesco Petrelli, Luca Pucci, Paola Bezzi

**Affiliations:** Department of Fundamental Neurosciences, Faculty of Biology and Medicine, University of Lausanne, rue du Bugnon 9, 1005 Lausanne, Switzerland

## Abstract

Astrocytes are highly secretory cells, participating in rapid brain communication by releasing glutamate. Recent evidences have suggested that this process is largely mediated by Ca^2+^-dependent regulated exocytosis of VGLUT-positive vesicles. Here by taking advantage of VGLUT1-pHluorin and TIRF illumination, we characterized mechanisms of glutamate exocytosis evoked by endogenous transmitters (glutamate and ATP), which are known to stimulate Ca^2+^ elevations in astrocytes. At first we characterized the VGLUT1-pHluorin expressing vesicles and found that VGLUT1-positive vesicles were a specific population of small synaptic-like microvesicles containing glutamate but which do not express VGLUT2. Endogenous mediators evoked a burst of exocytosis through activation of G-protein coupled receptors. Subsequent glutamate exocytosis was reduced by about 80% upon pharmacological blockade of the prostaglandin-forming enzyme, cyclooxygenase. On the other hand, receptor stimulation was accompanied by extracellular release of prostaglandin E_2_ (PGE_2_). Interestingly, administration of exogenous PGE_2_ produced *per se* rapid, store-dependent burst exocytosis of glutamatergic vesicles in astrocytes. Finally, when PGE_2_-neutralizing antibody was added to cell medium, transmitter-evoked exocytosis was again significantly reduced (by about 50%). Overall these data indicate that cyclooxygenase products are responsible for a major component of glutamate exocytosis in astrocytes and that large part of such component is sustained by autocrine/paracrine action of PGE_2_.

## 1. Introduction

The morphology and the location of astrocytes place them in a unique position to be able to listen and respond to neuronal activity [1–5]. Astrocytes express a wide variety of functional neurotransmitter receptors essential for sensing neuronal activity [6]. Many of these receptors are G-protein-coupled receptors (GPCRs) that, upon activation, stimulate phospholipase C and form inositol (1,4,5)-triphosphate (IP3) which increases the intracellular calcium (Ca^2+^) concentration through the release of Ca^2+^ from intracellular stores [6]. The intracellular cascade resulting in Ca^2+^ rise in astrocytes is the main mechanism these cells use to transduce synaptic activity. It is well established that the GPCR- mediated Ca^2+^ variations in astrocytes can trigger release of chemical substances [7, 8] such as excitatory amino acids (D-serine, glutamate) [2, 9, 10], ATP, and related nucleotides and nucleosides [11–13] or proinflammatory mediators such as eicosanoids (prostaglandins or PG) [2, 14] and tumor necrosis factor alpha (TNF*α*; [5, 15–17]). Interestingly, prostaglandin E_2_ (PGE_2_) and TNF*α* have been described to play an important role in the modulation of the regulated secretion of glutamate [5, 15–17]. PGE_2_ and TNF*α* at pathological concentrations appear to exert a potent control on Ca^2+^-dependent glutamate release from astrocytes [15, 18] and therefore could directly influence glial cells potentially resulting in complex changes in the brain network. Thus, when a local inflammatory reaction is triggered in the brain, the increased levels of such proinflammatory mediators can deeply alter the properties of glial network and thus of neuronal network [7]. However, PGE_2_ and TNF*α* are also present in the normal brain, albeit at much lower levels than during inflammatory reactions. Constitutive levels of TNF*α*, in particular, have been implicated in control of the stability of neuronal networks in response to prolonged changes in activity via the phenomenon of synaptic scaling [19, 20] and play a role in controlling the strength of excitatory synaptic transmission by promoting the insertion of AMPA receptors at the surface [21, 22]. The involvement of TNF*α* in regulating glutamate release from astrocytes during physiological conditions has been found in TNF*α*- and TNF receptor 1 knockout mice, pointing to a permissive role for the cytokine in the exocytosis of glutamate from astrocytes [17]. Recently, it has been discovered the way how TNF*α* modulates glutamate release from astrocytes and how this impinges on the astrocytic modulation of synaptic activity [5]. Much less information is available about the mechanism by which PGs can control glutamate release in response to activation of GPCRs [2, 15]. Here by taking advantage of a construct containing the vesicular glutamate transporter 1 and a pH-sensitive fluorescent marker of fusion (VGLUT1-pHluorin) and of total internal reflection fluorescence (TIRF) microscopy, we investigated the role of PGs in the glutamate exocytosis processes in astrocytes. We initially characterized secretory organelles expressing VGLUT1-pHluorin in astrocytes and found that the VGLUT1-pHluorin-expressing vesicles contain glutamate and belong to the family of small synaptic-like microvesicles (SLMVs) and not of other larger secretory organelles (such as dense core granules or lysosomes). Then we found that the exocytosis of such glutamatergic SLMVs, elicited by two endogenous mediators, as diverse as glutamate and ATP, are strongly depressed by pharmacological inhibition of cyclooxygenase (COX). We also provide evidence that PGE_2_ exerts most of its activity in amplifying exocytosis of glutamate after it is released in the extracellular medium. We conclude that activation of COX pathway should be regarded as a crucial step in the modulation of the GPCR mediated glutamate exocytosis from astrocytes.

## 2. Material and Methods

### 2.1. Pharmacological Agents, Constructs, and Transfection

All agents (acetylsalicylic acid, indomethacin, prostaglandin E2, adenosine 5′ triphosphate disodium salt (ATP), (+)-*α*-Methyl-4-carboxyphenylglycine (MCPG), adenosine-3′-phospho-5′-phosphosulfate (A3P5PS), 2-methylthioadenosine 5′ diphosphate trisodium salt (2MeSADP), and (S)-3,5-dihydroxyphenylglycine hydrate (DHPG)) were from Sigma (St. Louis, USA), unless otherwise indicated. 2-Methyl-6-(phenylethynyl)-pyridine (MPEP) and pyridoxalphosphate-6-azophenyl-2′,4′-disulfonic acid tetrasodium salt (PPADS) are from Tocris Cookson (Bristol, UK). Anti-PGE_2_ antibody (AbPGE_2_) was from Cayman Chemical (Liestal, Switzerland); cyclopiazonic acid (CPA, CalBiochem, USA); Alexa 568-conjugated transferrin (Life Technologies, USA); plasmid containing the VGLUT1-pHluorin constructs was prepared as previously described [24]. The plasmid (0.5 *μ*g for single transfection experiments) was transfected into primary rat cortical astrocytes cultures with FuGene6 (3 *μ*L, Roche Diagnostics, Switzerland).

### 2.2. Astrocyte Cultures for Imaging Experiments

Astrocyte cultures containing >99% GFAP-positive cells (≤8% of which were positive for the neural precursor marker LeX) were obtained from newborn rats. They were prepared as described [9], plated (2.5 × 10^4^ cells) on glass coverslip, and transfected 6–8-days later with VGLUT1-pHluorin. From 2 to 5 days after transfection, and coverslips were mounted in the open laminar flow perfusion incubator at 37°C (Harvard Apparatus, USA) on the stage of a Zeiss Axiovert 200 fluorescence inverted microscope modified for TIRF experiments (Visitron System, Germany). The experimental chamber (250 *μ*L volume) was perfused at a rate of 1–1.5 mL/min. The stimulus (DHPG, ATP, tACPD/AMPA, PGE_2_, 2MeSADP) was applied rapidly (2 sec) via a software-controlled microperfusion fast-step device (100 *μ*L/min, Warner Instruments Corp., USA). Cells were perfused at 37°C in a HEPES-KRH buffer containing (in mM) NaCl 120, KCl 3.1, MgCl_2_ 2, CaCl_2_ 1.8, NaH_2_PO_4_ 1.25, HEPES-Na 25 (buffered to pH 7.4), and glucose 4. In experiments with CPA, PPADS, A3P5PS, MCPG, MPEP, INDO, ASA, and AbPGE_2_ the drugs were diluted in HEPES-KRH and incubated for 15 min before the application of the stimulus.

### 2.3. Optical Imaging

TIRF illumination (TIRFi) was used for our experiments. The expanded beam of a 488/568 nm argon/krypton multiline laser (20 milliwatts, Laserphysics, Germany) passed through an AOTF laser wavelength selector (VisiTech International, UK) synchronized with a SNAP-HQ CCD camera (Roper Scientific, Germany) under Metafluor software (Universal Imaging, USA) control and was introduced to the coverslip from the high numerical aperture objective lens (Zeiss *α*-plan FLUAR 100X). Light entered the coverslip and underwent total internal reflection at the glass-cell interface. In our experimental conditions, penetration depth of TIRFi was calculated to be about 90 nm [17, 25]. In single-wavelength TIRFi experiments (488 nm) the laser beam was filtered via the Zeiss filter set 10 and images were acquired at 20–40 Hz (Zeiss, Switzerland). In dual-wavelength TIRF illumination (488/568 nm), laser beams were combined by a dichroic mirror from the Zeiss filter 24 at 20–40 Hz. The pixel size was 126 nm (at binning 2).

### 2.4. Image Analysis

Video images, digitized with MetaFluor, were analyzed with MetaMorph software (Universal Imaging, USA). The fusion events of VGLUT-pHluorin positive vesicles were manually selected and counted in areas of 6000 pixels on cell surface as already reported [9, 26, 27]. A fluorescent spot was counted as “fusion event” when the pHluorin fluorescence signal of a single SLMV increased over basal by ≥4-fold.

### 2.5. Immunocytochemistry

Astrocytes were plated on glass coverslips coated with 2 mg/mL poly-L-lysine and 33 mg/mL laminin and cultured for 2 days. The cells were rinsed with phosphate-buffered saline (PBS) and fixed in ice-cold methanol for 15 min. After two washes in ice-cold PBS, the coverslips were incubated for 10 min with PBS containing 0.5% saponin (PBS-S) and rinsed three times for 5 min with PBS. They were then incubated for 30 min in PBS-S containing 1% bovine serum albumin (BSA) and for 1 hour at room temperature in the presence of the primary antibodies diluted in PBS-S plus 1% BSA. The cells were rinsed with PBS, incubated 1 hour with the secondary antibody, and mounted for confocal microscopy (Leica SP5 AOBS Confocal Microscope). Primary antibodies were rabbit GFP (1 : 500, Chemicon), mouse VGLUT1 (1 : 500, Chemicon), VGLUT2 (1 : 2000, gift Robert Edwards, USA), VAMP3, (1 : 1000, Synaptic System), glutamate (1 : 3000, gift Vidar Gundersen, Oslo), VAMP2 (1 : 1000, Synaptic System), phogrin (1: 500, gift Romano Regazzi, Lausanne), LAMP1 (1 : 100, Calbiochem), EAA1 (1 : 100, BD Transduction Lab), and Tf receptor (1 : 100, Invitrogen). Secondary antibodies were Cy3 or FITC-conjugated (1 : 200, Molecular Probes).

### 2.6. Monitoring of Extracellular PGE_2_ Formation

Extracellular PGE_2_ was measured using a sensitive EIA kit (Prostaglandin E2 EIA kit-Monoclonal, Cayman Chemical Company, Ann Arbor). Cultured astrocytes plated on Petri dishes were washed twice with a KRH buffer containing (in mM) NaCl 120, KCl 3.1, MgCl_2_ 2, CaCl_2_ 1.8, NaH_2_PO_4_ 1.25, and HEPES-Na 25 (buffered to pH 7.4). Subsequently they were stimulated (3 min) with agents dissolved in the same buffer also containing an antiPGE_2_ antibody (AbPGE_2_, Cayman Chemical) at concentration buffering >1000 pg/mL PGE_2_. At the end of stimulations, the extracellular medium was rapidly collected, lyophilized and kept at −80°C until performing the EIA assay according to instructions.

### 2.7. Statistical Analysis

The experiments were analyzed using the SAS statistical package (SAS Inc., Cary, NC, USA). Statistical differences were tested by *t*-test and *P* values of 0.01** or 0.05*.

## 3. Results

**Figure 1:**
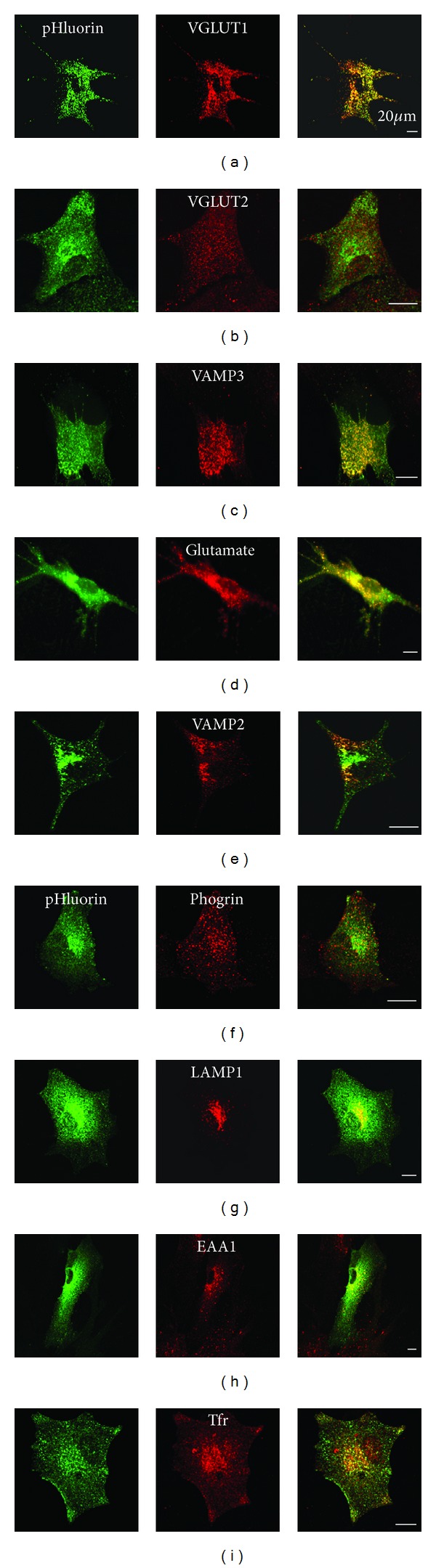
VGLUT1-pHluorin is mainly expressed on a specific population of glutamatergic synaptic like microvesicles. In the figure the left panels (in green) show astrocytes transfected with VGLUT1-pHluorin construct revealed by rabbit antibody against GFP. The middle panels (in red) show the markers of the intracellular secretory organelles, revealed by mouse antibodies against specific markers of ((a)–(e)) synaptic like microvesicles ((a) VGLUT1, (b) VGLUT2, (c) VAMP3, (d) glutamate, (e) VAMP2), of (f) dense core granules (phogrin), of (g) late endosomes, multivesicular bodies and lysosomes (LAMP1), of (h) early endosomes (EAA1), and of (i) recycling endosomes (transferrin receptor, Tfr). The right panels show the merged images. Bars: 20 *μ*m.

**Figure 2:**
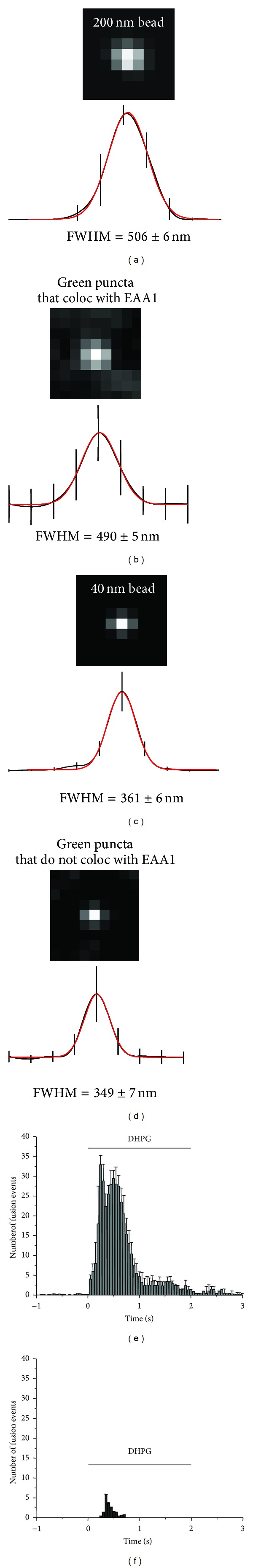
Analysis of VGLUT1-pHluorin vesicles that colocalize with markers of early or recycling endosomes. (a)–(d) Estimation of the size of vesicles expressing VGLUT1-pHluorin. Analysis of individual vesicle was performed in confocal images of VGLUT1-pHluorin-expressing astrocytes by plotting fluorescence intensity of pHluorin spots against distance from the centre of the spot (black curve ± SD). Such an analysis provided an estimation of the average fluorescence profile otherwise called “radial sweep” [23]. The fluorescence intensity values obtained from the radial sweep were well fitted by a one-dimensional Gaussian function (red curve). Such a curve represents the average radial sweep value obtained from 20 vesicles. Note that the half maximum value of pHluorin-expressing vesicle positive for EAA1 ((b), marker of early endosomes, 490 ± 5 nm) is similar to that of 200 nm fluorescent beads ((a), 506 ± 6 nm) and the half maximum value of pHluorin-expressing vesicle that do not express EAA1 ((d), 349 ± 7 nm) is similar to that of 40 nm fluorescent beads ((c), 361 ± 6 nm). (e), (f) Temporal distribution of VGLUT1-pHluorin and Alexa-Tf 568 fusion events evoked by DHPG application. (e) Each individual histogram represents the number (mean ± SD) of fusion events detected from VGLUT1-pHluorin vesicles in a 50 ms-long frame (*n* = 5 cells). (f) Fusion events (mean ± SD) detected from VGLUT1-pHluorin and Alexa-Tf568 double positive vesicles in the same cells as in (e). Each histogram represents the number of fusion events detected in a 50 ms-long frame (*n* = 5 cells).

Glutamatergic vesicles in astrocytes have been highlighted by transfecting cultured cells with the fluorescent construct VGLUT1-pHluorin, consisting of vesicular glutamate transporter 1 (VGLUT1) fused to a pH sensitive GFP mutant (pHluorin; [28]). Overexpression of VGLUT1-pHluorin in primary cortical astrocytes produced a punctate pattern of fluorescence (Figure 1). Astrocytes, similar to specialized secretory cells, contain three types of secretory organelles, the glutamate containing synaptic-like microvesicles (SLMVs) [9, 29, 30], the peptide containing large dense-core granules (LDCGs; [31, 32]), and the lysosomes [11–13]. These secretory organelles can be distinguished by immunocytochemistry and confocal analysis in primary cultured cells by using antibodies directed against endogenous markers [33]. In order to characterize which population of secretory organelles expressed VGLUT1-pHluorin, we performed a series of immunolabeling and confocal analysis. The VGLUT1-expressing vesicles were well colocalized with anti-VGLUT1 antibody (92 ± 3.5% for *n* = 7 cells, Figure 1(a)) but not with anti-VGLUT2 antibody (5 ± 2.7% for *n* = 5 cells, Figure 1(b)), indicating that VGLUT1-pHluorin is expressed on a particular set of intracellular glutamatergic vesicles. The VGLUT1-expressing vesicles showed a large co-localization with markers of SLMVs [9] such as VAMP3 (or cellubrevin, 94 ± 5.5% for *n* = 5 cells, Figure 1(c)) and glutamate (92 ± 6.2% for *n* = 5 cells, Figure 1(d)). Interestingly, the VGLUT1-associated vesicles showed only a small co-localization with endogenous VAMP2 (17 ± 2% for *n* = 5 cells, Figure 1(e)) and with markers of other secretory organelles such as LDCGs (phogrin, 2.3 ± 1.7% for *n* = 5 cells, Figure 1(f)) or lysosomes, (LAMP1, 3 ± 1.2% for *n* = 5 cells, Figure 1(g)). We also checked the co-localization of VGLUT1-positive vesicles with other lysosomal markers, including early endosomes with EAA1 (13 ± 7.3% for *n* = 5 cells, Figure 1(h)) [34] and the recycling endosomes with transferrin receptor (18 ± 7% for *n* = 5 cells, Figure 1(i), [35]). Early endosomes and recycling endosomes represent two distinct populations of endosomes that significantly colocalized with VGLUT1-pHluorin (co-localization about 20%). In order to clarify whether the organelles double positive for VGLUT1-pHluorin and the marker of early endosomes (EAA1) represent a population different from SLMVs, we estimated the average fluorescence profiles (radial sweep; [23]) of fluorescent vesicles from the double immunofluorescent labeling experiments shown in Figure 1(h). For analysis, we compared the half maximum values (FWHM) of the curve obtained from isolated green fluorescent dots representing VGLUT1-pHluorin which colocalize with EAA1 with the corresponding value of radial sweep curves of fluorescent beads of different diameters (40 nm and 200 nm). We found that the VGLUT1-pHluorin dots colocalized with EAA1 had a FWHM value similar to that of 200 nm beads (490 ± 5 nm, *n* = 20; 506 ± 6 nm, *n* = 20, resp., Figures 2(a) and 2(b)). Conversely, the green fluorescent dots of VGLUT1-pHluorin that do not colocalize with EAA1 had FWHM comparable to that of fluorescent beads of 40 nm of diameter (349 ± 7 nm and 361 ± 6 nm, resp., *n* = 20 for each, Figures 2(c) and 2(d)). Thus, the organelles double positive for pHluorin and EAA1 were clearly a different population of organelles from those expressing only VGLUT1-pHluorin and most probably represent part of VGLUT1-pHluorin-positive organelles undergoing endosomal/lysosomal recycling pathway. In a parallel set of experiments, in order to rigorously determine whether population of VGLUT1-pHluorin positive vesicles also positive for marker of recycling endosomes was able to undergo regulated exocytosis, we monitored the exocytosis processes evoked by an agonist of group I metabotropic glutamate receptors (mGluR), dihydroxyphenylglycine (DHPG) [9, 29]. Primary astrocytes transfected with VGLUT1-pHluorin and preincubated with a specific marker of recycling endosomes (Alexa 568-conjugated transferrin, [35]) have been challenged with DHPG (100 *μ*M) for 2 seconds (s). The VGLUT1-pHluorin- and the Alexa568-positive vesicles have been followed in real time with the dual wavelength TIRF experiments (488 nm and 568 nm laser TIRF). Two seconds of DHPG application evoked a burst of exocytosis of VGLUT1-pHluorin vesicles as previously reported (Figure 2(e); [9]) and only 16% of fusion events of VGLUT1-pHluorin/Alexa-568 double positive vesicles (Figure 2(f)). Overall, these data showed that VGLUT1-pHluorin can be used as a surrogate marker for glutamatergic SLMVs in astrocytes.

**Figure 3:**
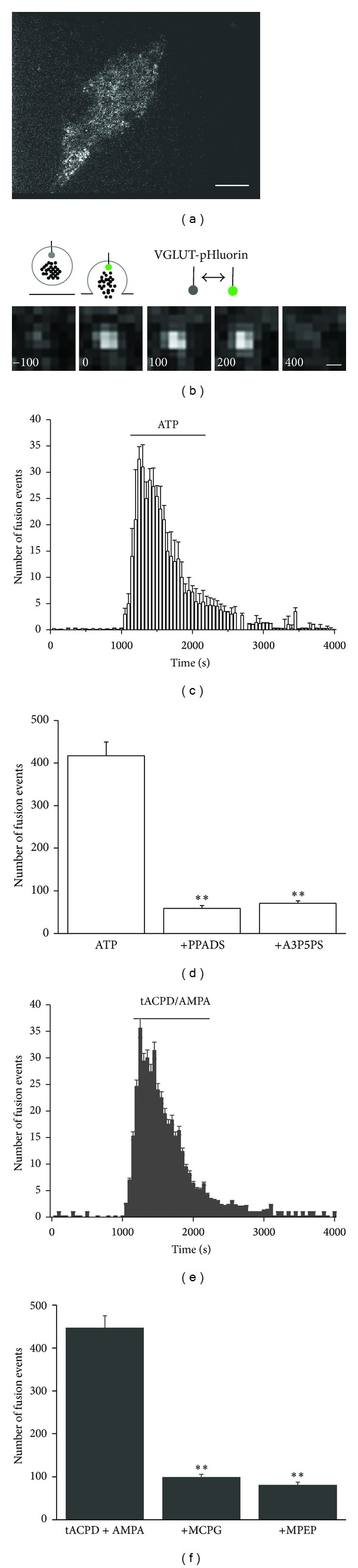
Pharmacological characterization of the receptor subtypes mediating exocytosis of VGLUT1-pHluorin positive vesicles in response to ATP and glutamate agonists. (a) TIRF image showing an astrocyte transfected with VGLUT1-pHluorin. Bar 20 nm. (b) Stereotyped sequence of pHluorin destaining reveals exocytosis of a VGLUT1-pHluorin positive vesicle. The sequential gray scale micrographs represent the fate of pHluorin before (−100 ms) and during (100, 200, 400 ms) the fusion event. Bars: 380 nm. The scheme shows the behaviour of pHluorin before and after fusion event. Note that the color code for the pHluorin fluorescence signal is gray when the signal is off and green when it is on. (c), (d) P2Y_1_ receptors mediate the ATP-evoked exocytosis. (c) Temporal distribution of fusion events evoked by ATP (100 *μ*M). (d) Histograms represent the total number of fusion events evoked by ATP (417.14 ± 32.4) that is strongly inhibited in the presence of the P2 purine antagonists PPADS (100 *μ*M, 58.6 ± 7) as well as of the P2Y_1_-selective compound, A3P5PS (100 *μ*M, 70.2 ± 5.8). Data are ± SEM of 4 cells. (e), (f) mGluR5 mediates the response to t-ACPD, in the presence of AMPA. (e) Temporal distribution of fusion events evoked by 50 *μ*M t-ACPD+50 *μ*M AMPA. (f) Histograms represent the total number of fusion events evoked by t-ACPD+AMPA (447.1 ± 28.7) that is strongly inhibited in the presence of the mGluR antagonists, including the subtype-nonselective MCPG (500 *μ*M, 98.3 ± 7.4) and the mGluR5-selective MPEP (200 nM, 80.1 ± 7). Data are ± SEM of 4 cells. Statistical significance of inhibition with receptor antagonists was calculated using *t*-test (**P < 0.01).

To analyze the role of prostaglandins (PGs) in the DHPG-evoked exocytosis of glutamatergic vesicles in astrocytes, we started by monitoring single exocytic events of VGLUT1-pHluorin-associated vesicles (Figures 3(a) and 3(b); [9]), evoked by two distinct protocols of receptor stimulation. When either purinergic receptor or glutamatergic receptor agonists were locally administrated (ATP, 100 *μ*M, or coapplication of t-ACPD and AMPA each at 50 *μ*M), rapid burst of exocytosis was elicited (Figures 3(c) and 3(e)), suggesting that the two stimulation protocols shared similar excitation-secretion coupling mechanism. The nature of the receptors activated by the three protocols of stimulation was then investigated by pharmacological agents with known selectivity. The response to ATP was abolished by pretreatment with PPADS (−86%, 100 *μ*M), an agonist of most P2 purinergic receptors, as well as with A3P5PS (−83%, 100 *μ*M), a selective P2Y_1_ antagonist (Figure 3(d); [36]). Consistent with these results, 2MeSADP, a P2Y_1_ agonist, potently stimulated glutamate exocytosis (data not shown), (see Supplementary Figure 1; in Supplementary Material available online at http://dx.doi.org/10.1155/2014/254574 [5, 17]), suggesting that P2Y_1_ receptors are the predominant mediators of the glutamate exocytosis action of ATP. The presence of P2Y_1_ receptors in astrocytes and their role in Ca^2+^ signaling pathway leading to modulation of synaptic activity have recently been reported [5, 37]. As for glutamate, it has been established that the potent glutamate releasing effect of t-ACPD+AMPA in astrocytes mainly depends on the simultaneous activation of group I mGluRs and ionotropic receptors of the AMPA-preferring subtype [2]. Here, we confirm that the response to t-ACPD+AMPA (each at 50 *μ*M) is reduced to less than 40% by administration of MCPG (−78%, 500 *μ*M), a nonselective mGluR antagonist, and by MPEP (−82%, 200 nM), a specific antagonist selective for mGluR5 (Figure 3(f)). On the whole, pharmacological profile identified mGluR5 as the metabotropic receptor subtype that is implicated in the glutamate release response to glutamatergic stimulation. Consistent with these results, DHPG, an agonist of the group I of mGluR, evoked glutamate exocytosis in astrocytes [9, 29]. The two receptors here identified as mediators of the exocytosis of glutamatergic vesicles in astrocytes belong to the G protein-coupled family, which are known to be expressed in astrocytes and to release Ca^2+^ from internal stores via IP3 pathway [38].

**Figure 4:**
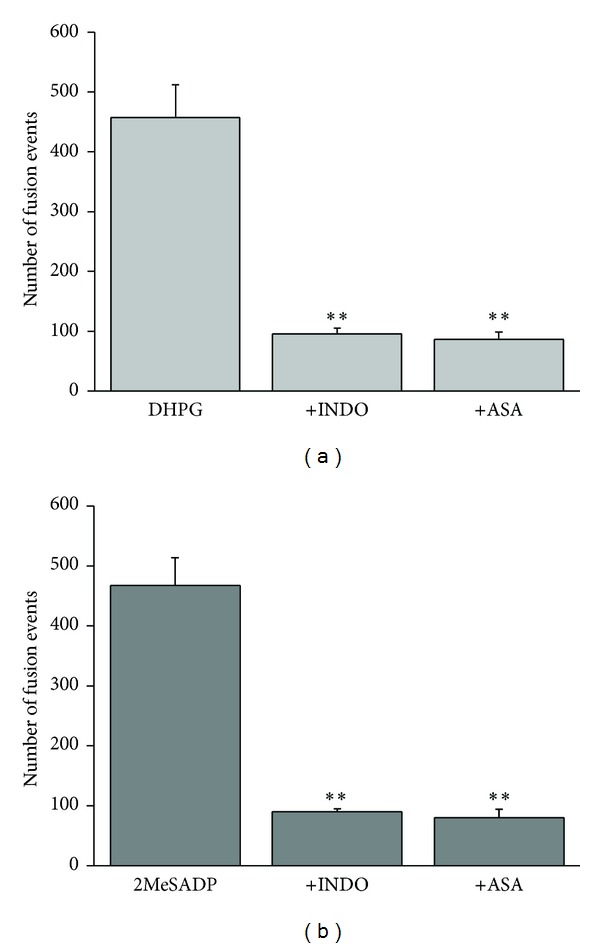
COX blockers strongly inhibit the exocytosis of glutamate evoked by activation of group I mGluR and of purinergic P2Y_1_ receptor. (a), (b) Quantitative histograms represent the total number of fusion events evoked by either DHPG (100 *μ*M, 456.7 ± 54.8) or 2MeSADP (20 *μ*M, 467.6 ± 46.8) in the presence of COX blockers, INDO (1 *μ*M; 94.9 ± 9.6, 88.8 ± 5.4, resp.) or ASA (10 *μ*M, 86.4 ± 11.7, 79.5 ± 14.4, resp.). Data are ± SEM of 4 cells. Statistical significance of inhibition with receptor antagonists was calculated using *t*-test (**P < 0.01).

Glutamatergic stimulation of astrocytes is known to promote rapid, phospholipase A2-dependent activation of the arachidonic acid cascade [2, 39]. Pharmacological inhibition of the different eicosanoid-forming pathways indicates that cyclooxygenase (COX) but not other arachidonate metabolic enzymes is involved in the mechanism leading to glutamate release [2, 15, 17]. COX is an enzyme that is responsible for the formation of prostanoids [40]. The three main groups of prostanoids (prostaglandins, prostacyclins, and thromboxanes) are each involved in the inflammatory response but prostaglandins (PGs) can control release of glutamate from astrocytes. We studied the involvement of PGs in the DHPG-evoked exocytosis of glutamatergic SLMVs by preincubating cells with two COX inhibitors, indomethacin (INDO, 1 *μ*M) and aspirin (ASA, 10 *μ*M). We found that the two anti-inflammatory drugs decreased by about 80% the releasing effect of DHPG and of 2MeSADP (Figures 4(a) and 4(b)). This evidence is consistent with a scenario in which the excitation-secretion pathway leading to glutamate exocytosis from astrocytes is controlled, at some level, by the COX pathway.

**Figure 5:**
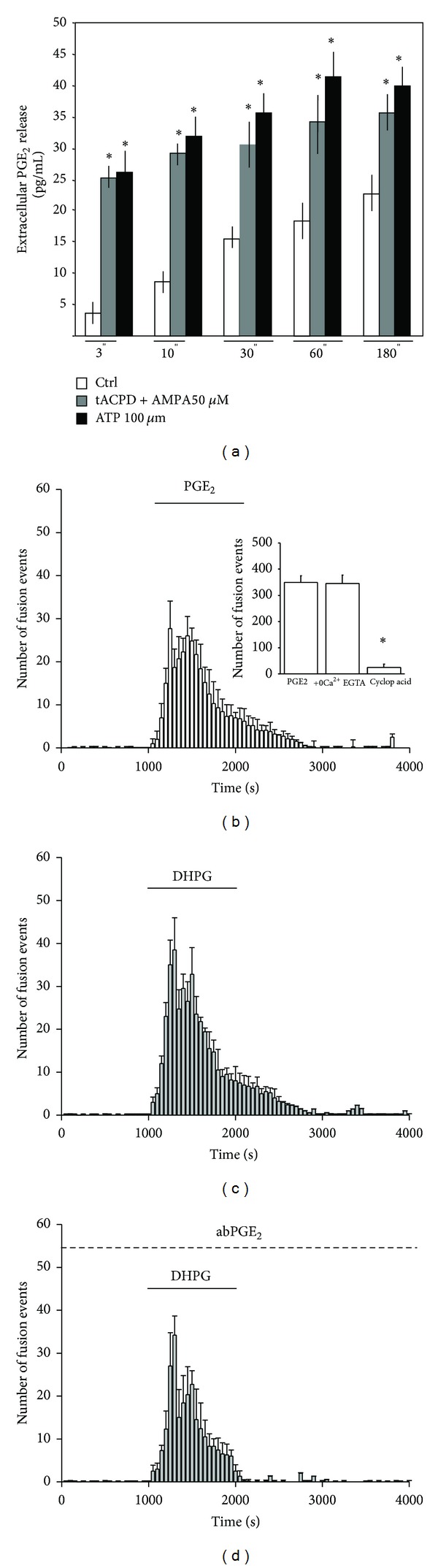
Extracellular PGE_2_: accumulation in response to various stimuli and effects on exocytosis of glutamatergic vesicles. (a) Extracellular accumulation of PGE_2_ (expressed as pg/mL) in response to 3 min stimulation with either t-ACPD+ AMPA (each at 50 *μ*M) or ATP (100 *μ*M). Each point represents the average ± SEM of two experiments in triplicate with each stimulus. (b) Temporal distribution of fusion events evoked by PGE_2_ (50 *μ*M). Inset histograms represent the total number of fusion events evoked by PGE_2_ (349 ± 26) in the presence of 0 mM Ca^2+^ and 5 mM of EGTA (345 ± 32) or cyclopiazonic acid (CPA, 10 *μ*M, 25 ± 12). (c) Temporal distribution of fusion events evoked by DHPG (100 *μ*M). (d) Inhibitory effect of AbPGE_2_ (buffering capacity >1000 pg/mL PGE_2_) on exocytosis of glutamatergic vesicles evoked by DHPG (100 *μ*M). Histograms represent temporal distribution of fusion events evoked by DHPG in the presence of AbPGE_2_. Statistical significance was calculated using *t*-test (*P < 0.05).

In light of the above results, we wanted to examine the mechanism by which COX inhibitors decreased glutamate exocytosis in astrocytes. It is well known that a COX product, prostaglandin E_2_ (PGE_2_), is formed and released in extracellular space after activation of mGluRs [2] and that it causes by itself intracellular Ca^2+^ elevations leading to glutamate release in astrocytes [2, 14]. Interestingly, we also found that the time course analysis of PGE_2_ accumulation in response to activation of glutamatergic or purinergic receptors was almost maximal within the first 3 seconds of stimulation (Figure 5(a); [2, 17]). Therefore, the kinetics of the PGE_2_ release is fully compatible with a potential role of this COX metabolite in rapid cell signaling. We wanted to investigate the effect of PGE_2_ on the intracellular pathways leading to exocytic burst of glutamatergic SLMVs. We found that administration of PGE_2_ (50 *μ*M) to astrocytes caused a rapid burst of exocytosis that did not depend on Ca^2+^ influx from outside of the cells but on Ca^2+^ release from internal store (Figure 5(b)). In fact incubation with cyclopiazonic acid (1 *μ*M), which causes depletion of Ca^2+^ from internal stores by blocking SERCA, abolished the response to PGE_2_ (Figure 5(b) inset). Since PGE_2_ is released upon receptor activation and promotes* per se* Ca^2+^ elevations and glutamate release, we specifically investigated whether extracellular PGE_2_ contributes to the physiological response of astrocytes to glutamate exocytosis. We blocked extracellular PGE_2_ with a specific antibody (AbPGE_2_).  Figure 5(d) shows that when AbPGE_2_ was present in the bath, a condition in which PGE_2_ is rapidly and efficiently sequestered (AbPGE_2_ buffering capacity is >1000 pg/mL PGE_2_), DHPG- evoked exocytosis of glutamatergic SLMVs was significantly reduced (−43 ± 12%, *n* = 6 cells). Similar results were obtained for 2MeSADP (Supplementary Figure 1(b); −51 ± 14%, *n* = 4 cells, resp.). Interestingly, the action of AbPGE_2_ was specific and could not be reproduced by the boiled protein (not shown). Therefore, the whole body of evidence suggests that extracellular PGE_2_ accounts for a significant component of mGluR5- and P2Y_1_- dependent exocytosis of glutamatergic SLMVs in astrocytes.

## 4. Discussion

Astrocytes play an important role in the integration of rapid chemical signaling in the brain [41]. They function as signal integrators, since they generate outputs with variable timing in response to particular signals received from surrounding neuronal cells to communicate with the same neurons and/or with other cellular components of the brain circuits. A crucial element that facilitates the integrating functions of astrocytes is the regulated exocytosis of chemical substances [9, 29, 42–44]. By this process, astrocytes exert modulatory influences on neighboring cells and are thought to participate in the control of synaptic circuits and cerebral blood flow [45, 46]. Exocytosis is an evolutionary trait of eukaryotic cells that leads in a given secretory cell to a release of chemical content by a fast mechanism into the extracellular space and thus to communication with neighboring cells. In neurons, exocytosis represents one of the fastest biological events known. Similar to neurons or neurosecretory cells, astrocytes express at least three different secretory organelles: the small synaptic like microvesicles (SLMVs) [29, 30, 47], the large dense core granules (LDCGs) [31, 32, 48] which store and release distinct cargo, and lysosomes [49, 50]. In neurons and specialized secretory cells, these organelles have specialized physiological functions, are typically found in different regions of the cell and are regulated by different intracellular signaling pathways of calcium. Interestingly, in our immunolabeling and confocal analysis performed in cultured astrocytes we found that small VAMP3-, VGLUT1-, and glutamate-positive SLMVs, large phogrin-positive DCGs, and lysosomes positive for markers of multivesicular bodies and late endosomes or early and recycling endosomes exist in the same astrocytes. Our results confirmed that the task of identifying a specific population of secretory organelles in astrocytes is very difficult. Moreover, studying properties of exocytosis constitutes a challenge because the cell biological basis of this process is very incompletely defined in these cells. For example, researchers have only recently started to understand that astrocytic exocytosis relies on multiple populations of secretory vesicles, which calls for the definition of adequate criteria to recognize the distinct populations and study them in isolation. In fact, most of the early studies of astrocyte exocytosis could not distinguish such heterogeneity as they used generic exocytosis reporters (such as synthetic fluorescent dyes). Thus, these studies did not allow an accurate description of the dynamics and properties of astrocytic secretion because they mixed contributions by more than one exocytic organelle population. As a result, the types of vesicles used in Ca^2+^-regulated exocytosis in astrocytes are under debate. A good experimental strategy involves trying to specifically live-stain a selected population (e.g., by transfecting the cells with a population-specific fluorescent reporter). This approach must, however, be validated by subsequent co-localization analysis with markers of the intracellular organelles, which will provide additional useful information on the nature of the stained organelles. By combining TIRF experiments and transfection of a fluorescent tool used to visualize exo/endocytosis processes in living neurons (VGLUT1-pHluorin) [28], we visualized VGLUT1-positive SLMVs. We therefore characterized vesicles expressing VGLUT1 and found that they have diameter similar to that of fluorescent beads of 40 nm, contain glutamate, and express at least one of the VAMP proteins necessary for regulated exocytosis (VAMP3).

Once confirmed that VGLUT1-pHluorin was a correct tool for studying glutamatergic SLMVs in astrocytes, we then investigated the activity of the two endogenous mediators that participate in brain intercellular chemical communication: glutamate and ATP. Glutamate and ATP are well-recognized brain signaling agents that are released in response to neuronal or glial cell stimulation and that mediate intercellular signaling [7, 51]. Independently of their origin, both glutamate and ATP were previously shown to activate astrocytes causing intracellular Ca^2+^ increase followed by glutamate release [2, 52, 53]. The Ca^2+^-dependent exocytosis of glutamate in astrocytes is controlled by multiples mediators, all acting via receptors that belong to the GPCR super family stimulating IP3 productions (P2Y_1_ for ATP, mGluR5 for glutamate). Stimulation of GPCR also promoted stimulation of the COX pathway of the arachidonic acid metabolism with the ensuing production and release of PGs being critical for the full efficiency of the excitation-secretion coupling in astrocytes. We in fact found that COX inhibition caused a dramatic reduction in the total number of fusion events of glutamatergic SLMVs caused by activation of both purinergic and glutamatergic receptors. By analyzing the temporal distribution of the exocytic events, we found an intriguing aspect of the mode of action of PGs. Inhibition of the PGs by either COX inhibitors or a neutralizing antibody specific for PGE_2_ produced a significant modification of the temporal characteristics of the exocytic burst. In particular, while the fast initial component (0–400 ms) was most often left intact, the second component of the burst (500 ms–1.6 s) was often suppressed or significantly slowed down, suggesting that PG-mediated signaling is heavily implicated in this later, slower phase of the release. Interestingly, the rapid phase of the exocytic burst was sustained almost exclusively by “resident” vesicles, whereas the slow phase was mainly sustained by “newcomer” vesicles [9]. This duality is reminiscent of observations previously done in neurons where only readily releasable synaptic vesicles are rapidly recycled and reused [54]. As we do not know whether the “newcomer” and the “resident” vesicles represent distinct population of SLMVs, it is possible that the “newcomers” may indeed represent the same population of “resident” vesicles that undergoes a second round of exocytosis upon rapid recycling. In this context PGE_2_ seemed to amplify the second round of exocytosis. Since it is known that PGE_2_ is immediately released from astrocytes upon stimulation [2, 17] and that exogenous PGE_2_ induced rapid, Ca^2+^-dependent burst of glutamatergic SLMVs, it follows that a component of the burst of exocytosis in response to glutamate or ATP requires an autocrine/paracrine action of PGE_2_. The comparison effects of the COX blockers (about −80%) and AbPGE_2_ (about −45%) also indicate that extracellular PGE_2_ cannot be solely responsible for all the observed COX-dependent events. At present we do not have a specific explanation for these differences. It is possible that other PGs could be released together with PGE_2_. For instance, it has been shown that intracellular Ca^2+^ elevations in astrocytes are known to stimulate the production of a full range of COX derivates [55] and some of them have been shown to cause Ca^2+^ rises and glutamate release from astrocytes [2, 56]. The present data confirm and extend our previous finding that PGE_2_ participates to glutamate-dependent cell-cell communication [2], suggesting a physiological function for this mediator in the intact brain. The autocrine/paracrine action of PGE_2_ may thus participate in the spatial control of astrocytic signal propagation. Therefore, it is possible that alterations in PGs synthesis during brain inflammations and other pathological states have a profound impact on the chemical communication in the brain. PGE_2_ stimulation, for instance, acts downstream of CXCL12, as well as TNF*α* pathways. Although both these pathways have been suggested to be involved in the physiological glutamate-mediated brain signalling [5, 26], it is likely that PGE_2_ synthesis will be largely amplified during inflammatory conditions, following the fate of its upstream inflammatory messengers [18]. As the whole pathway increases glutamate release from astroglial cells, much higher levels of PGE_2_ might represent the ultimate messenger leading to pathological increase of extracellular glutamate levels.

## Supplementary Material

Activation of purinergic P2Y_1_ receptors with a specific agonist 2MeSADP (20 *μ*M) stimulates glutamate exocytosis as measured by number of fusion events (Suppl. fig. 1a). This can be reduced (by about 50%) by pre-incubating astrocytes with an anti-PGE_2_ antibody (Suppl. fig. 1b) thus supporting the DHPG data (Fig. 5)
