# Supplementary material for: G-Protein Coupled Receptor-Evoked Glutamate Exocytosis from Astrocytes: Role of Prostaglandins
Source: Neural Plast. 2014 Jan 16;2014:254574. doi: 10.1155/2014/254574 (PMC3914554; doi:10.1155/2014/254574)
Supplement: Supplementary file 1 — Activation of purinergic P2Y1 receptors with a specific agonist 2MeSADP (20 μM) stimulates glutamate exocytosis as measured by number of fusion events (Suppl. fig. 1a). This can be reduced (by about 50%) by pre-incubating astrocytes with an anti-PGE2 antibody (Suppl. fig. 1b) thus supporting the DHPG data (Fig. 5) [file 254574.f1.pdf]

Supplementary Fig 1

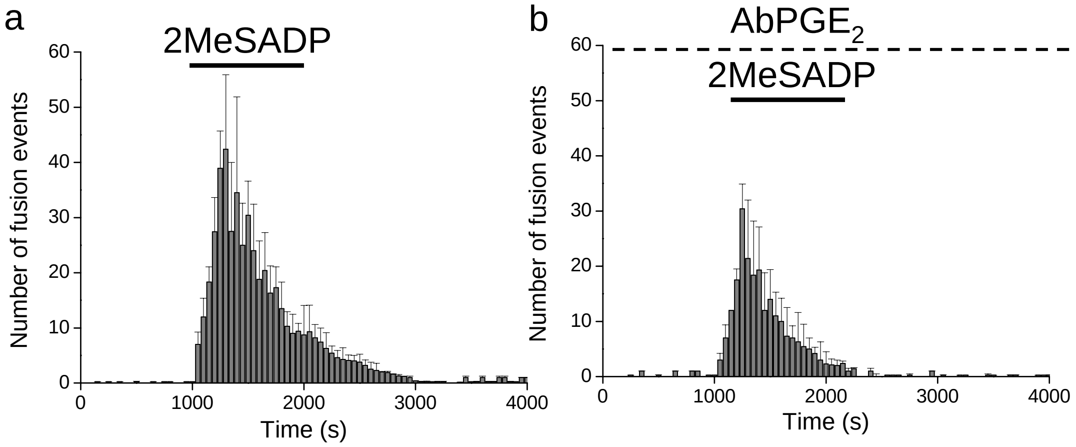

Fig 1. a. Temporal distribution of fusion events evoked by 2MeSADP (20 μM). b. Inhibitory effect of $AbPGE_2$ (buffering capacity >1000 pg/ml PGE_2) on exocytosis of glutamatergic vesicles evoked by 2MeSADP (20 μM). Histograms represent temporal distribution of fusion events evoked by 2MeSADP in the presence of $AbPGE_2$.
